# Home Transcutaneous Electrical Stimulation Rehabilitation Program for Patients With Ankylosing Spondylitis: Crossover Trial

**DOI:** 10.2196/58048

**Published:** 2024-10-28

**Authors:** Yu-Chih Lin, Chen-Ching Wu, Wan-Yu Sung, Jeng-Hsien Yen, Yi-Ching Lin

**Affiliations:** 1 Division of Rheumatology Department of Internal Medicine Kaohsiung Medical University Hospital Kaohsiung Taiwan; 2 Division of General Internal Medicine Department of Internal Medicine Kaohsiung Medical University Hospital Kaohsiung Taiwan; 3 Department of Medical Humanities and Education College of Medicine Kaohsiung Medical University Kaohsiung Taiwan; 4 Graduate Institute of Clinical Medicine College of Medicine Kaohsiung Medical University Kaohsiung Taiwan; 5 Institute of Biomedical Sciences National Sun Yat-Sen University Kaohsiung Taiwan; 6 Department of Laboratory Medicine Kaohsiung Medical University Hospital Kaohsiung Taiwan; 7 Department of Laboratory Medicine School of Medicine, College of Medicine, Kaohsiung Medical University Kaohsiung Taiwan; 8 Doctoral Degree Program of Toxicology College of Pharmacy, Kaohsiung Medical University Kaohsiung Taiwan

**Keywords:** ankylosing spondylitis, galvanic response, home medical device, rehabilitation, transcutaneous electrical stimulation

## Abstract

**Background:**

Maintaining physical function and preserving spinal flexibility have been challenging in managing ankylosing spondylitis (AS). Most rehabilitation programs, including manual therapy, massage, hydrotherapy, and acupuncture, cannot be performed at home. The effect of transcutaneous electrical nerve stimulation (TENS) was validated in treating AS, but no home TENS system has explored its efficacy to date.

**Objective:**

This study aims to evaluate the efficacy of a home TENS system with a novel treatment program for patients with AS.

**Methods:**

The modified WeHeal TS-200 TENS and galvanic response system provided home-based TENS treatment for patients with AS. Patients were divided into a 2-month course group and a 1-month course group. After the first treatment course, patients went through a washout period for the same duration of their treatment course. Participants could decide whether to accept the second course of treatment. The Bath Ankylosing Spondylitis Disease Activity Index (BASDAI), Bath Ankylosing Spondylitis Functional Index (BASFI), Schober test, finger-to-floor flexion test, enthesis score, cytokines, chemokines, inflammatory factors, and immunoglobulins were measured to evaluate its efficacy. The clinical trial protocol (1096607481) received approval from the Ministry of Health and Welfare in Taiwan.

**Results:**

A total of 9 patients (5 in a 2-month course group and 4 in a 1-month course group) completed the first treatment course, and 5 patients (4 in a 2-month course group and 1 in a 1-month course group) completed the sequential treatment course. The weighted results showed that patients reported an improving BASFI score (mean difference –0.9, SD 1.7; *P*=.03) after treatment. Looking into the trajectories, declined BASFI and BASDAI scores were noticed during treatments; this score increased during the washout period. There were improving trends in the Schober test (mean difference 1.9, SD 4.9; *P*=.11) and finger-to-floor flexion test (mean difference –0.6, SD 9.5; *P*=.79), but the results were not statistically significant. The response of cytokines, chemokines, inflammatory factors, and immunoglobulins before and after treatment did not show a consistent trend, and all results were not statistically significant (all *P*>.05).

**Conclusions:**

The home TENS device demonstrated a potential role in AS management. It may improve accessibility and adherence for patients with AS and provide remote monitoring for clinicians. Further research can compare the effectiveness of electrotherapy at home or in a medical setting and focus on integrating the home TENS system and exercise program to enhance patients’ physical functions and spinal flexibility.

## Introduction

Ankylosing spondylitis (AS), a subset of prevalent axial spondylarthritis, is an inflammatory disorder mainly affecting the axial skeleton and sacroiliac joints [1] and affects approximately 0.1% and 1.4% of the population, predominantly young and middle-aged adults [2-4]. The prevalence is 2 to 3 times higher in men than women [5,6]. Moreover, patients with AS often have physical restrictions, which result in sick leave, unemployment, and heavy social burden [7].

Treatment goals for AS include reducing symptoms, improving and maintaining spinal flexibility, reducing functional limitations, maintaining the ability to work, and decreasing the complications associated with the disease [1]. Therefore, in addition to pharmacological interventions, active exercise and rehabilitation programs are essential to maintain physical activity in patients with AS. For example, manual therapy, massage, hydrotherapy, electrotherapy, and acupuncture have been adopted as rehabilitation programs for AS [8]. The patient’s functional status and quality of life can be preserved using these approaches [9,10]. The combination of transcutaneous electrical nerve stimulation (TENS) and balance and postural stability exercises have synergic effects on improving physical function and self-reported symptoms [11].

TENS and electroacupuncture have been widely used in patients with AS to control pain, improve physical functions, and enhance mobility [12,13]. Although TENS has been proposed as an effective treatment for AS since 1984 [14,15], the settings of electrical stimulation, including waveform, pulse frequency, and pulse width, still mainly rely on the experience of doctors and physical therapists. Therefore, programming and feasibility are critically important in the daily treatment of patients with AS. However, the information on effective protocols for AS is limited.

Most importantly, patients with AS receive TENS and electroacupuncture predominantly in clinics and hospitals, which may lead to poor adherence and efficacy. To our knowledge, no previous clinical trial focused on the home electrical stimulation rehabilitation program for patients with AS. Therefore, the study aims to evaluate the efficacy of an innovative home electrical stimulation rehabilitation program in AS.

## Methods

### Clinical Trial Device and Treatment Protocol

**Figure 1:**
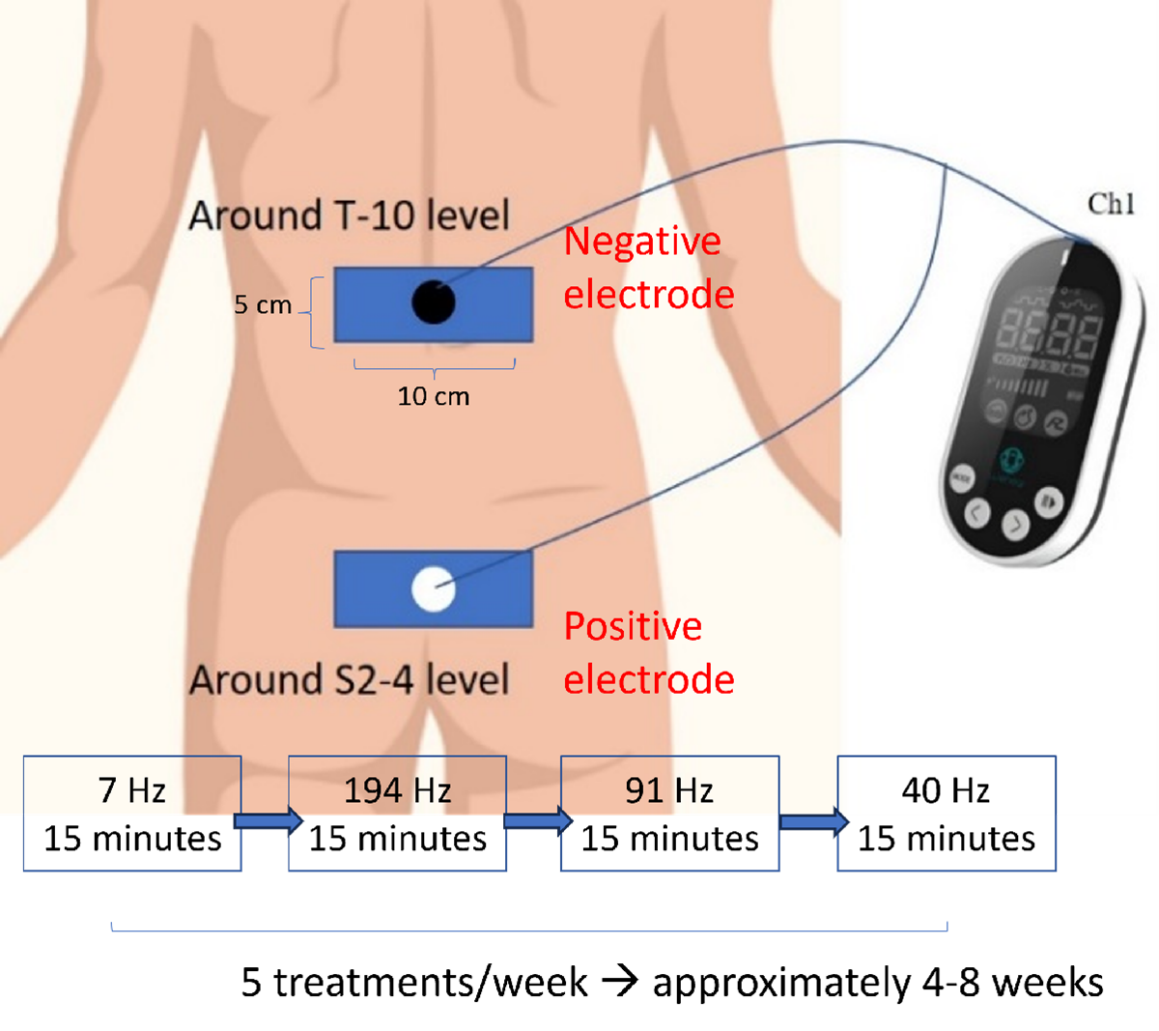
The scheme of WeHeal TENS and galvanic response system. TENS: transcutaneous electrical nerve stimulation.

WeHeal TS-200 is a class-II home medical device approved by the Taiwan Food and Drug Administration (TFDA), with 4 classified functions: TENS, electrical muscle stimulation, galvanic response, and bioelectrical impedance analysis. The device’s standard hardware features monophasic and biphasic square waves, with a frequency range of 1-200 Hz and a wave width spanning 80-440 Hz. In this trial, we modified the device’s TENS and galvanic response system to treat AS and record the galvanic response of the patients, respectively. We reprogrammed the firmware of the device and designed a preconfigured 60-minute treatment module—monophasic square wave; wave width: 200 s; and pulse frequency: 7 Hz (15 minutes), 194 Hz (15 minutes), 91 Hz (15 minutes), and 40Hz (15 minutes). The design of the right-skewed, bell-curved pulse frequency course stemmed from the previous literature, and the selected frequency is related to the healing frequency of “spine,” “fibrosis,” “anti-inflammation,” and “back pain” [16,17]. The size of the electrode pad (square, 5×10 cm) was designed to cover the average spine width. One electrode pad connected to the white electrode (positive electrode) was placed around the S2-4 level, and the other electrode pad connected to the black electrode (negative electrode) was placed around the T-10 level (Figure 1). The current intensity was gradually increased to the individual tolerance of patients, which did not trigger pain. Before and after each treatment, the reprogrammed firmware commanded the device to measure the bioimpedance between 2 electrodes in predicting the condition of patients’ lower back. The modified device for this trial stored the number of treatments the patient received in 4 weeks (5 treatments a week) and each galvanic response (100 Hz biphasic square wave; measuring voltage 1.98 Vp-p) data in its memory. Next, the device transmitted data via Bluetooth to the research assistant’s phone when patients returned to the clinic. Finally, the data were sent to doctors’ computers (Figure S1 in Multimedia Appendix 1).

### Study Design

The study recruited patients diagnosed with AS aged between 20 and 70 years, all of whom experienced chronic pain persisting for more than 3 months and had a visual analog pain scale score greater than 3. Patients with pacemakers or who had received steroid therapy, joint injections, or TENS as part of a rehabilitation program were excluded from the study.

Potential participants were initially screened by rheumatologists during outpatient visits. Following this preliminary screening, the trial researcher conducted a detailed evaluation to confirm whether these cases met the study’s inclusion criteria. Informed consent was obtained from each participant before their inclusion in the study. Participants were divided into a 2-month course group and a 1-month course group. Upon completing the initial treatment course, participants underwent a washout period of the same duration as their treatment course. After the washout period, participants could choose whether to proceed with a second course of treatment. Measurements were taken at 4 time points: upon enrollment, after the first treatment course, after the washout period, and after the second treatment course (Figure S2 in Multimedia Appendix 1). During the trial, participants did not receive other exercise or rehabilitation programs. The clinical trial protocol received approval from the Ministry of Health and Welfare in Taiwan (TFDA clinical trial: 1096607481).

### Covariates

We collected basic demographics from patients, including age, sex, time interval from the AS diagnosis date to the index date, comorbidities (diabetes mellitus, hyperlipidemia, sciatica, asthma, arthropathy, psychological disorders, and iridocyclitis), and concurrent medications (cyclooxygenase-2 inhibitors, muscle relaxants, and sulfasalazine).

### Outcomes

Using measurements in 4 domains, we evaluated the program’s efficacy by evaluating the changes in self-rating scores, physical examinations, blood tests, cytokines, and inflammatory biomarkers. Self-rating scores comprised the Bath Ankylosing Spondylitis Disease Activity Index (BASDAI) and Bath Ankylosing Spondylitis Functional Index (BASFI) [3,18]. The BASDAI and BASFI scores ranged from 0 to 10. Higher scores indicated more functional limitations and higher disease activity. The physical examination focused on the Schober test [19], the finger-to-floor flexion test [20], and the enthesis score [18]. Increasing Schober test results represented the increasing lumbar spine range of motion. By contrast, lower finger-to-floor flexion test and enthesis scores indicated improving clinical condition.

Immunoglobulin (Ig) levels were evaluated, including IgG, IgA, and IgM [21]. Serum cytokines and chemokines including interleukin-1ß (IL-1ß), IL-3, IL-17F, IL-22 [22,23], monocyte chemoattractant protein 1 [24], and monodansylcadaverine were tested [25]. Other inflammatory factors, such as erythrocyte sedimentation rate and C-reactive protein [26], were measured to represent AS disease activity. The results of galvanic response in each treatment were recorded in the level of skin impedance (Ω) between 2 electrodes. Lower impedance levels represented higher conductance, which indicated muscle and fascia relaxation.

### Statistical Analyses

Descriptive statistics were used to assess patient demographics, number and percentage for categorical variables, and the mean (SD) and median for continuous variables. Wilcoxon signed ranked test was conducted for paired results before and after TENS treatment [26]. Statistical analyses were conducted using SAS (version 9.4; SAS Institute). A *P* value of <.05 was considered statistically significant.

### Ethical Considerations

The study followed the ethical guidelines of the Declaration of Helsinki and received approval from the institutional review boards of Kaohsiung Medical University Hospital (approval: KMUHIRB-F(I)-20200093; date: June 12, 2020). All participants gave their implied consent to take part in the study. Each participant received a subsidy of NTD 5000 (US $155). They were informed that their participation was voluntary and that they could choose to withdraw at any time. To ensure the legitimate use of research data and safeguard the rights and interests of the participants, the data exclusively served the purpose of this research. Throughout the study period, data were anonymized using codes to protect the privacy of the participants. The data were securely stored on a dedicated computer or hard drive and maintained in strict confidentiality. Access to the data was controlled by the principal investigator through relevant passwords.

## Results

### Clinical Demographics of Participants

First, 10 patients were recruited and 1 was excluded. Consequently, 9 patients (male: n=6, 67%; female: n=3, 33%) were enrolled in the first course of treatment. The average interval from the diagnosis date to the index date was 3.3 (SD 2.6; median 3.5, IQR 3.6) years (Table 1). The average number of treatments for the first course was 33.0 (SD 8.8; median 31, IQR 11.0; maximum 48). Eight (89%) out of 9 patients concurrently took cyclooxygenase-2 inhibitors, two-thirds (n=6, 67%) took sulfasalazine, and 4 (45%) took muscle relaxants.

Next, 5 patients continued the second course of treatment. The average number of treatments in the second course decreased to 15.3 (SD 3.9; median 15.3, IQR 7.0; maximum=21) and 77% of the recommended treatments. In sum, 14 treatment courses were completed in this study.

**Table 1:** Basic characteristics of the study population (n=9).

Characteristics			Values
**Age** **(years)**			
	Mean (SD)	34.1 (8.8)	
	Median (IQR)	31.5 (14.8)	
**Interval (years; from diagnosis date to the index date)**			
	Mean (SD)	3.3 (2.6)	
	Median (IQR)	3.5 (3.6)	
**Sex, n (%)**			
	Male	6 (67)	
	Female	3 (33)	
**Comorbidity, n (%)**			
	Diabetes mellitus	1 (11)	
	Hyperlipidemia	3 (33)	
	Sciatica	2 (22)	
	Asthma	2 (22)	
	Arthropathy	1 (11)	
	Psychological disorder	1 (11)	
	Iridocyclitis	1 (11)	
**Concurrent medications, n (%)**			
	Cyclooxygenase-2 inhibitors	8 (89)	
	Muscle relaxants	4 (44)	
	Sulfasalazine	6 (67)	

### Changes in Clinical Evaluations Before and After TENS Treatment

The weighted results showed that patients had improved self-rating scores after treatments, particularly the BASFI score (mean difference –0.9, SD 1.7; *P*=.03; Table 2). Separately, the BASFI score showed significant improvement after a 1-month course (mean difference –0.5, SD 0.6; *P*=.03), while the result showed no statistical significance after a 2-month course (mean difference –1.3, SD 2.0; *P*=.50; Table 3). In general, there were improving trends in the Schober test (mean difference 1.9, SD 4.9; *P*=.11) and finger-to-floor flexion test (mean difference –0.6, SD 9.5; *P*=.79), but the results were not statistically significant.

**Table 2:** The weighted changes of clinical evaluations before and after treatment courses (n=19)^a^.

		Precourse value			Postcourse value			Difference^b^			*P* value^c^
		Mean (SD)	Median (IQR)	Mean (SD)		Median (IQR)	Mean (SD)		Median (IQR)		
**Self-rating score**											
	BASDAI^d^ scale	3.7 (2.3)	3.0 (2.4)	2.7 (2.8)		1.9 (4.5)	–1.0 (2.4)		–1.5 (1.6)	.08	
	BASFI^e^ scale	1.8 (2.2)	0.8 (3.4)	0.8 (1.3)		0.6 (0.4)	–0.9 (1.7)		–0.2 (1.7)	.03^c^	
**Physical examination**											
	Schober test (cm)	4.6 (3.8)	5.0 (7.0)	6.5 (3.5)		6.0 (2.5)	1.9 (4.9)		2.0 (4.3)	.11	
	Finger-to-floor flexion test (cm)	13.1 (14.2)	10.0 (13.0)	12.5 (9.0)		11.0 (12.5)	–0.6 (9.5)		0 (5.5)	.79	
	Enthesis score	1.7 (2.3)	1.0 (2.0)	1.9 (3.1)		0 (3.0)	0.2 (2.6)		0 (3.0)	.73	
**Inflammatory factors and immunoglobulins**											
	IgG^f^	1299.9 (294.4)	1230.0 (300.0)	1307.1 (324.4)		1280.0 (480.0)	7.2 (124.6)		0 (130.0)	.8	
	IgA	212.3 (69.6)	227.0 (127.0)	202.9 (57.9)		221.0 (97.0)	–9.3 (31.1)		–3.0 (26.0)	.21	
	IgM	91.0 (73.8)	79.1 (74.0)	94.6 (83.7)		72.7 (87.3)	3.7 (15.3)		0.6 (18.1)	.31	
	ESR^g^	16.4 (11.9)	13.0 (13.0)	14.7 (12.4)		12.0 (23.0)	–1.7 (4.8)		–2.0 (6.0)	.15	
	CRP^h^	5.1 (4.4)	4.5 (6.4)	4.3 (2.1)		4.0 (4.4)	–1.3 (3.2)		–0.4 (4.5)	.09	
**Cytokines and chemokines**											
	IL-1ß^i^ (n=18)	9.0 (14.3)	5.9 (6.6)	10.9 (15.4)		4.7 (13.8)	1.8 (10.5)		–0.3 (15.2)	.48	
	IL-3 (n=18)	293.8 (843.6)	7.8 (197.2)	399.6 (1210)		4.9 (208.0)	105.9 (368.6)		–0.3 (3.3)	.25	
	IL-17F (n=18)	3736.0 (11,741)	197.4 (236.7)	4035.3 (12,627)		205.8 (394.4)	299.2 (892.8)		4.1 (110.0)	.18	
	IL-22 (n=18)	16.8 (14.3)	13.7 (8.0)	16.5 (14.8)		12.1 (9.4)	–0.3 (18.4)		0.3 (6.0)	.95	
	IL-28 (n=18)	105.3 (190.6)	8.3 (182.8)	109.0 (210.3)		3.1 (171.8)	3.7 (24.7)		0 (5.2)	.54	
	IL-34 (n=18)	49.1 (125.9)	3.9 (20.8)	62.8 (178.0)		2.0 (27.3)	13.7 (53.4)		–2.0 (2.6)	.30	
	CCL-2 (MCP-1^j^; n=18)	169.3 (294.5)	95.6 (67.9)	195.1 (444.6)		57.9 (123.1)	25.8 (154.7)		0.7 (57.1)	.49	
	CCL-22 (MDC^k^; n=18)	625.3 (296.8)	587.2 (318.4)	600.0 (286.7)		583.8 (403.4)	–25.3 (242.7)		–78.5 (142.6)	.67	

^a^The 1-month course was used as the weighting basis, and the 2-month course weighed 2. In sum, this study included nine 1-month courses and five 2-month courses, resulting in 19 weighted courses.

^b^Difference was calculated by the postcourse value minus the precourse value in pairs.

^c^*P* value was calculated by paired 2-tailed *t* test for weighted paired results; *P*<.05.

^d^BASDAI: Bath Ankylosing Spondylitis Disease Activity Index.

^e^BASFI: Bath Ankylosing Spondylitis Functional Index.

^f^Ig: immunoglobin.

^g^ESR: erythrocyte sedimentation rate.

^h^CRP: C-reactive protein.

^i^IL: interleukin.

^j^MCP-1: monocyte chemoattractant protein 1.

^k^MDC: monodansylcadaverine.

**Table 3:** Self-rating scores and physical examinations among patients before and after 1-month or 2-month courses.

			2-month course (n=5)																			1-month course (n=9)																	
			Precourse value					Postcourse value					Difference^a^					*P* value^b^			Precourse value							Postcourse value					Difference^a^						*P* value^b^
			Mean (SD)		Median (IQR)		Mean (SD)			Median (IQR)		Mean (SD)			Median (IQR)					Mean (SD)				Median (IQR)			Mean (SD)			Median (IQR)		Mean (SD)			Median (IQR)				
**Self-rating score**																																							
	BASDAI^c^ scale	3.7 (1.5)		3.7 (1.9)		2.7 (2.6)			2.4 (1.6)		–1.0 (2.5)			–1.8 (0.9)		.63			3.7 (2.4)						3.0 (2.2)	2.6 (2.5)			1.2 (4.5)		–1.0 (1.7)			–1.1 (1.1)		.10			
	BASFI^d^ scale	1.9 (2.0)		0.8(3.2)		0.5 (0.2)			0.6 (0.1)		–1.3 (2.0)			–0.2 (3.4)		.50			1.7 (2.0)						1.0 (1.4)	1.2 (1.6)			0.5 (0.8)		–0.5 (0.6)			–0.5 (0.9)		.03^c^			
**Physical examination**																																							
	Schober test (cm)	4.0 (3.8)		5.0 (7.0)		6.3 (3.0)			6.0 (2.5)		2.3 (3.1)			3.0 (1.0)		.19			5.2 (2.8)						6.5 (3.5)	6.7 (3.3)			5.7 (1.5)		1.5 (5.4)			–1.0 (2.8)		≥.99			
	Finger-to-floor flexion test (cm)	14.2 (14.5)		9.0 (3.0)		12.3 (6.6)			9.0 (12.0)		–1.9 (11.5)			–1.0 (3.5)		.81			11.8 (10.7)						10.0 (15.0)	12.7 (9.4)			14.0 (14.0)		0.9 (2.8)			1.0 (5.0)		.43			
	Enthesis score	1.0 (1.0)		1.0 (2.0)		0.8 (1.3)			0 (1.0)		–0.2 (2.2)			–1.0 (3.0)		.94			2.4 (2.5)				2.0 (4.0)			3.1 (3.3)			2.0 (7.0)		0.7 (2.4)				0 (1.0)		.38		

^a^Difference was calculated by the postcourse value minus the precourse value in pairs.

^b^*P* value was calculated by Wilcoxon signed ranked test for paired results; *P*<.05.

^c^BASDAI: Bath Ankylosing Spondylitis Disease Activity Index.

^d^BASFI: Bath Ankylosing Spondylitis Functional Index.

**Figure 2:**
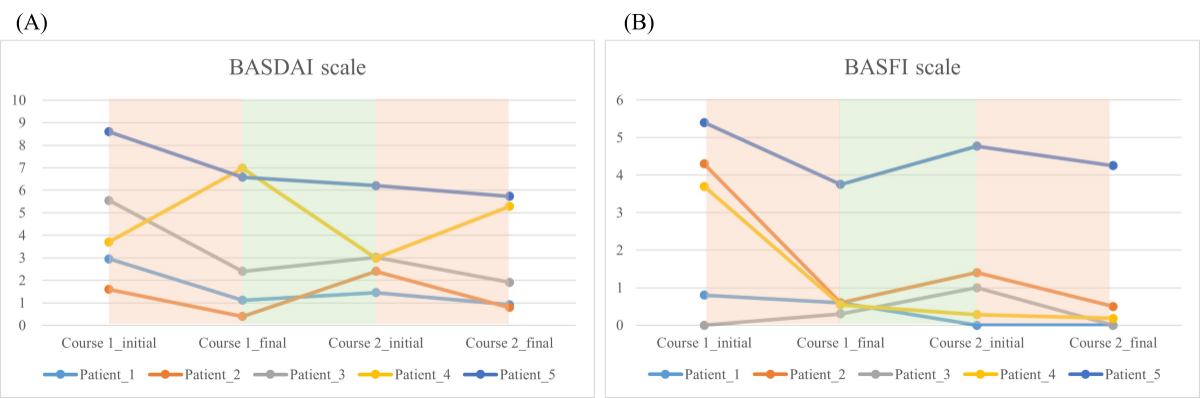
Trajectory changes of self-rating scales among 5 participants. (A) BASDAI and (B) BASFI scores. BASDAI: Bath Ankylosing Spondylitis Disease Activity Index; BASFI: Bath Ankylosing Spondylitis Functional Index.

**Figure 3:**
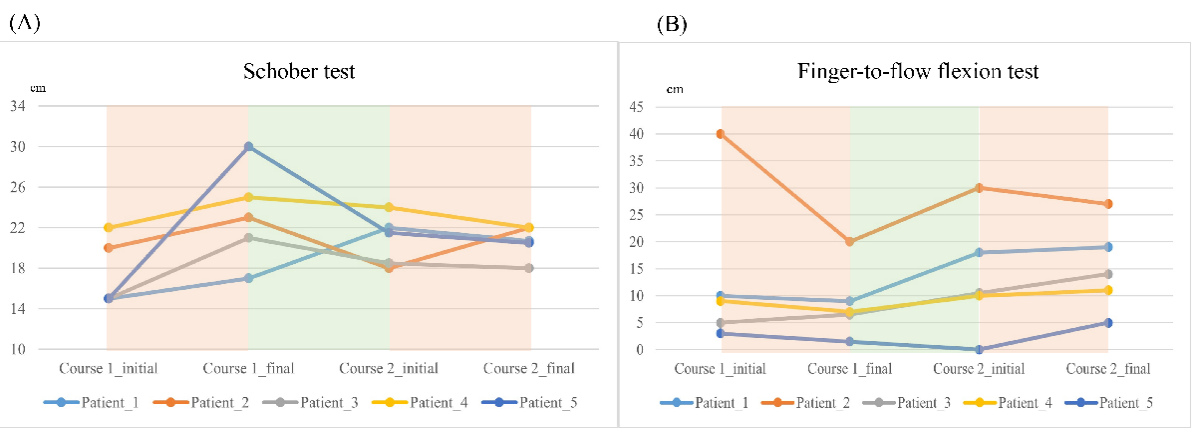
Trajectory changes of physical functions among 5 participants. (A) Schober test and (B) finger-to-floor flexion test.

Looking into trajectories, 4 (80%) out of 5 patients reported a declined BASDAI score during treatments; this score increased during the washout period (Figure 2A). The BASFI score shared a similar trend (Figure 2B). These trajectories indicated that TENS treatment benefited patients with AS in terms of BASDAI and BASFI scores. In the first treatment course, all patients performed better on Schober test, but the response in the second course was not evident (Figure 3A). Otherwise, only patient 2 markedly improved the finger-to-floor flexion test (Figure 3B).

The response of immune factors, cytokines, chemokines, and inflammatory factors before and after TENS treatment did not show a consistent trend, and all results were not statistically significant (all *P*>.05; Table 2). Moving to the results of galvanic response, we can notice the changes before and after each treatment (Figure S3 in Multimedia Appendix 1), which may be a good indicator of the effect of TENS treatments in patients with AS.

## Discussion

### Principal Findings

This trial revealed that the home TENS system was acceptable to patients with AS and had a high potential to be an effective rehabilitation program. Adherence to the treatment was good, and patients took 165% (33/20) and 77% (15.3/20) of the recommended number of treatments in the first and second courses, respectively. Moreover, the data on galvanic response showed that it is a good biomarker for evaluating the effect of each treatment. The physician can easily monitor these treatments every visit and read the feedback.

Overall, patients in this trial reported a significantly improved BASFI score after treatment. Although patients reported a more remarkable improvement in BASFI score in the 2-month course than in the 1-month course (mean difference –1.3, SD 2.0 vs –0.5, SD 0.6), the statistics showed no significance because of the small case number in the 2-month group. The result showed a similar trend in the Schober test—a mean difference of 2.3 (SD 3.1) and 1.5 (SD 5.4) in 2-month and 1-month courses, respectively. We suppose the longer the treatment lasts, the better the treatment outcome. Looking into trajectories, declined BASFI and BASDAI scores were noticed during treatments; this score increased during the washout period. Our results suggested that this system can improve the quality of life and maintain spinal flexibility among patients with AS.

Although the response of immune factors, cytokines, and inflammatory biomarkers before and after treatment did not show a consistent trend, and all results were not statistically significant, it has provided a reference for further research.

### Comparison With Prior Work

Although electrotherapy, such as TENS and electroacupuncture, has been widely used in current clinical practice for AS treatment, there is limited evidence from clinical trials to support its efficacy. Gemignani et al [15] conducted a double-blind study—20 patients received 10 treatments (pulse frequency of 5 Hz; 20 minutes each time) over 3 weeks [15]. Significant differences in pain and self-reported stiffness over the treatment period were found only in the treatment group. The result was consistent with the open study by Nienhuis and Hoekstra [14]. Based on our study, the trajectory analysis of the BASDAI score supported that electrical stimulation attenuated pain and stiffness during the treatment period, and the symptoms rebounded during the washout period.

According to a retrospective study, patients in the TENS group (n=36; pulse frequency of 100 Hz, pulse width of 100 µs, 30 minutes each time, 2 times weekly) did not show significant improvement in pain reduction, BASFI score, and quality of life after a 6-week treatment [13]. In this study, the median number of treatments for patients in each course was 31 (IQR 11.0; maximum 48; first course) and 15.3 (IQR 7.0; maximum=21; second course). Our study showed that the BASFI score decreased significantly after treatment, indicating an improved functional status. From our perspective, the unsatisfied outcomes from the previous study might stem from the insufficient treatment numbers.

### Strength and Limitations

This is the first clinical trial to evaluate the home-based electrical stimulation rehabilitation program for patients with AS. The main strength of this study was the comprehensive evaluation of its efficacy using self-rating scores, physical examinations, blood tests, cytokines, and inflammatory biomarkers. Based on a home-based, modified WeHeal TS-200 system, researchers, in particular, could monitor patient’s adherence and response to each treatment. In general, patients prefer to receive treatment at home. An effective home treatment program can improve accessibility, enhance adherence, and reduce patient time and cost. However, there were some limitations, such as the case number being relatively small. Second, 4 (44%) out of 9 patients did not undergo the washout period and second treatment course, resulting in most statistics only showing the trend but not the significant difference. Third, the effectiveness of treatment and the records of galvanic response may be influenced by the decay of electrode pads. Therefore, comparing measurement data before and after each treatment is more clinically meaningful. On the contrary, comparing values measured by galvanic response between multiple treatments may interfere with the decay of electrode pads. Finally, patients’ adherence declined in the second treatment course, which may interfere with the outcomes. However, this condition is common in real-world situations.

### Conclusions

The home TENS system in this trial improves self-reported health, maintains physical function, and preserves spinal flexion. It may improve accessibility and adherence for patients with AS and provide remote monitoring for clinicians. Further research can compare the effectiveness of electrotherapy at home or in a medical setting. Researchers can also focus on integrating home electrotherapy and exercise programs to enhance patients’ physical functions and spinal flexibility.

## Appendix

Multimedia Appendix 1 Supplementary figures.

## Abbreviations

AS: ankylosing spondylitis
BASDAI: Bath Ankylosing Spondylitis Disease Activity Index
BASFI: Bath Ankylosing Spondylitis Functional Index
Ig: immunoglobulin
IL: interleukin
TENS: transcutaneous electrical nerve stimulation
TFDA: Taiwan Food and Drug Administration

## Data Availability

The datasets generated and analyzed during this study are not publicly available due to privacy or ethical restrictions but are available from the corresponding author upon reasonable request.

## References

[1] Taurog JD, Chhabra A, Colbert RA (2016). Ankylosing spondylitis and axial spondyloarthritis. N Engl J Med.

[2] Zhao J, Huang C, Huang H, Pan JK, Zeng LF, Luo MH, Liang GH, Yang W, Liu J (2020). Prevalence of ankylosing spondylitis in a Chinese population: a systematic review and meta-analysis. Rheumatol Int.

[3] Chen YH, Huang WN, Chen YM, Lai KL, Hsieh TY, Hung WT, Lin CT, Tseng CW, Tang KT, Chou YY, Wu YD, Huang CY, Hsieh CW, Chen YJ, Liao YW, Chen HH (2022). The BASDAI cut-off for disease activity corresponding to the ASDAS scores in a Taiwanese cohort of ankylosing spondylitis. Front Med.

[4] Boonen A, van der Linden SM (2006). The burden of ankylosing spondylitis. J Rheumatol Suppl.

[5] Braun J, Sieper J (2007). Ankylosing spondylitis. Lancet.

[6] Masi AT, Wilkins WR (1996). Does male:female sex ratio in ankylosing spondylitis change with age?. J Rheumatol.

[7] Boonen A, Brinkhuizen T, Landewé R, van der Heijde D, Severens JL (2010). Impact of ankylosing spondylitis on sick leave, presenteeism and unpaid productivity, and estimation of the societal cost. Ann Rheum Dis.

[8] Passalent LA (2011). Physiotherapy for ankylosing spondylitis: evidence and application. Curr Opin Rheumatol.

[9] Durmuş D, Alayli G, Uzun O, Tander B, Cantürk F, Bek Y, Erkan L (2009). Effects of two exercise interventions on pulmonary functions in the patients with ankylosing spondylitis. Joint Bone Spine.

[10] Pécourneau V, Degboé Y, Barnetche T, Cantagrel A, Constantin A, Ruyssen-Witrand A (2018). Effectiveness of exercise programs in ankylosing spondylitis: a meta-analysis of randomized controlled trials. Arch Phys Med Rehabil.

[11] Gunay SM, Keser I, Bicer ZT (2018). The effects of balance and postural stability exercises on spa based rehabilitation programme in patients with ankylosing spondylitis. J Back Musculoskelet Rehabil.

[12] Zhang D, Liu W, Yang H, Wu Y (2016). Clinical review of ankylosing spondylitis treated with acupuncture and medicine. Zhongguo Zhen Jiu.

[13] Chen FC, Jin ZL, Wang D (2018). A retrospective study of transcutaneous electrical nerve stimulation for chronic pain following ankylosing spondylitis. Medicine.

[14] Nienhuis RL, Hoekstra AJ (1984). Transcutaneous electronic nerve stimulation in ankylosing spondylitis. Arthritis Rheum.

[15] Gemignani G, Olivieri I, Ruju G, Pasero G (1991). Transcutaneous electrical nerve stimulation in ankylosing spondylitis: a double-blind study. Arthritis Rheum.

[16] McMakin C (2017). The Resonance Effect: How Frequency Specific Microcurrent is Changing Medicine. 1st Edition.

[17] Jan MY, Bau J, Hsiu H, Chao PT, Wang WK, Lin Y (2006). A study on the wave propagation property III: With loop and microcirculation.

[18] Zochling J (2011). Measures of symptoms and disease status in ankylosing spondylitis: ankylosing spondylitis disease activity score (ASDAS), ankylosing spondylitis quality of life scale (ASQoL), bath ankylosing spondylitis disease activity index (BASDAI), bath ankylosing spondylitis functional index (BASFI), bath ankylosing spondylitis global score (BAS-G), bath ankylosing spondylitis metrology index (BASMI), dougados functional index (DFI), and health assessment questionnaire for the spondylarthropathies (HAQ-S). Arthritis Care Res.

[19] Yen YR, Luo JF, Liu ML, Lu FJ, Wang SR (2015). The anthropometric measurement of Schober's test in normal Taiwanese population. Biomed Res Int.

[20] Viitanen JV, Kautiainen H, Suni J, Kokko ML, Lehtinen K (1995). The relative value of spinal and thoracic mobility measurements in ankylosing spondylitis. Scand J Rheumatol.

[21] Kinsella TD, Espinoza L, Vasey FB (1975). Serum complement and immunoglobulin levels in sporadic and familial ankylosing spondylitis. J Rheumatol.

[22] Zochling J, Braun J (2005). Management and treatment of ankylosing spondylitis. Curr Opin Rheumatol.

[23] Ranganathan V, Gracey E, Brown MA, Inman RD, Haroon N (2017). Pathogenesis of ankylosing spondylitis—recent advances and future directions. Nat Rev Rheumatol.

[24] Kragstrup TW, Andersen T, Holm C, Schiøttz-Christensen B, Jurik AG, Hvid M, Deleuran B (2015). Toll-like receptor 2 and 4 induced interleukin-19 dampens immune reactions and associates inversely with spondyloarthritis disease activity. Clin Exp Immunol.

[25] Ma C, Wen B, Zhang Q, Shao P, Gu W, Qu K, Shi Y, Wang B (2019). Polydatin regulates the apoptosis and autophagy of fibroblasts obtained from patients with ankylosing spondylitis. Biol Pharm Bull.

[26] Divine G, Norton HJ, Hunt R, Dienemann J (2013). Statistical grand rounds: a review of analysis and sample size calculation considerations for Wilcoxon tests. Anesth Analg.

