# Supplementary material for: Home Transcutaneous Electrical Stimulation Rehabilitation Program for Patients With Ankylosing Spondylitis: Crossover Trial
Source: JMIR Form Res. 2024 Oct 28;8:e58048. doi: 10.2196/58048 (PMC11555451; doi:10.2196/58048)
Supplement: Multimedia Appendix 1 [file formative_v8i1e58048_app1.pdf]

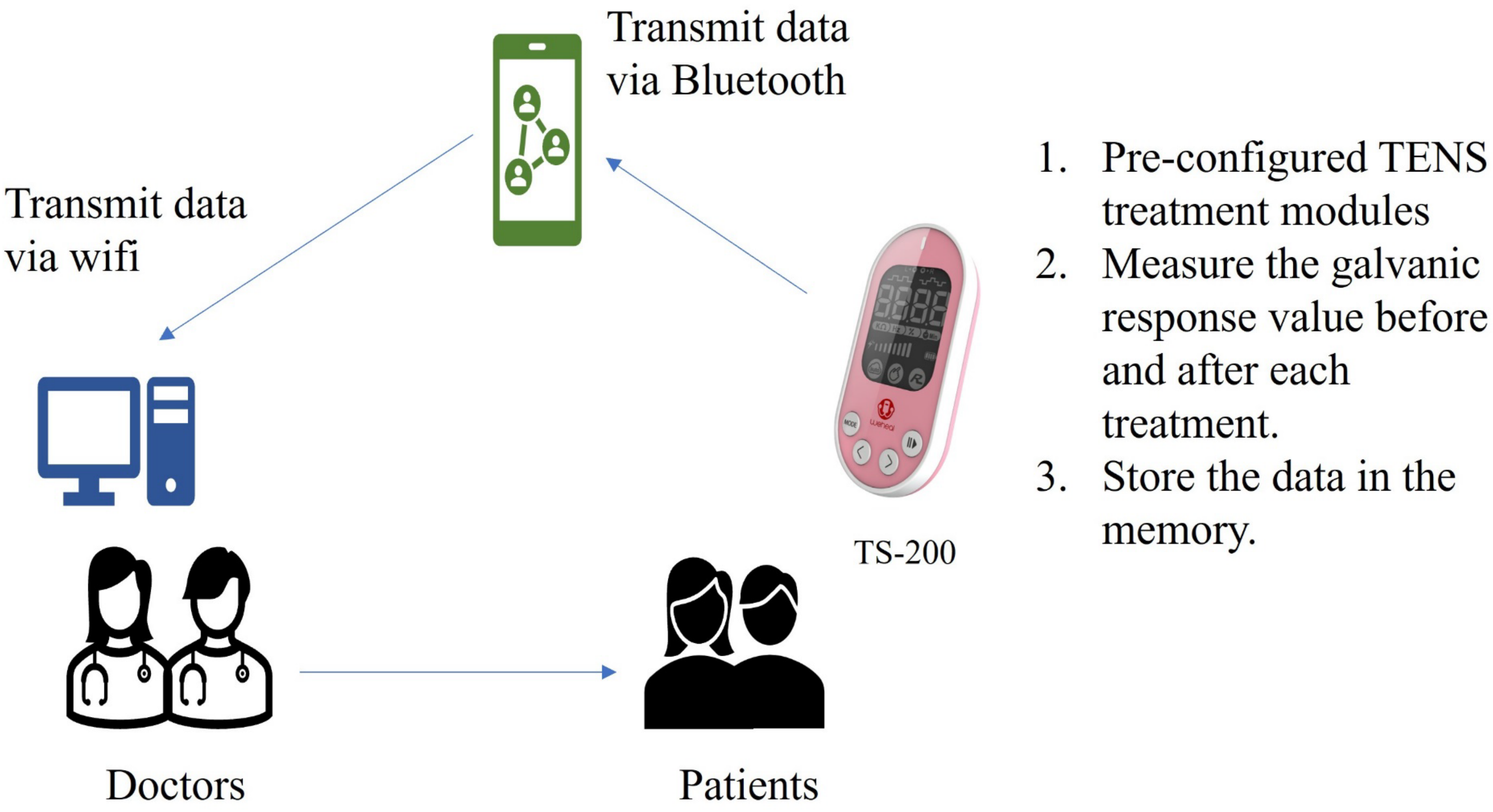

Figure S1. The scheme of how the galvanic response value data from the WeHeal® transcutaneous nerve stimulation (TENS) was collected from the devices to the researcher.

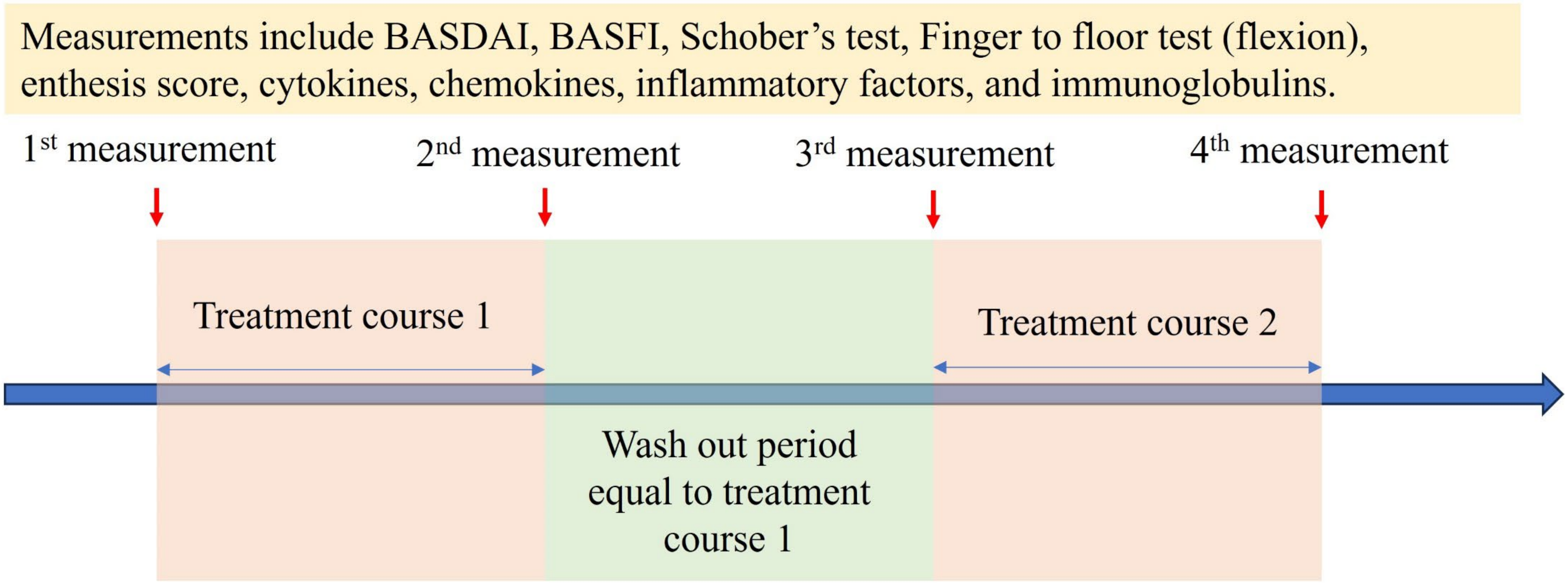

Figure S2. Cross-over study design. BASDAI, Bath Ankylosing Spondylitis Disease Activity Index; BASFI, Bath Ankylosing Spondylitis Functional Index

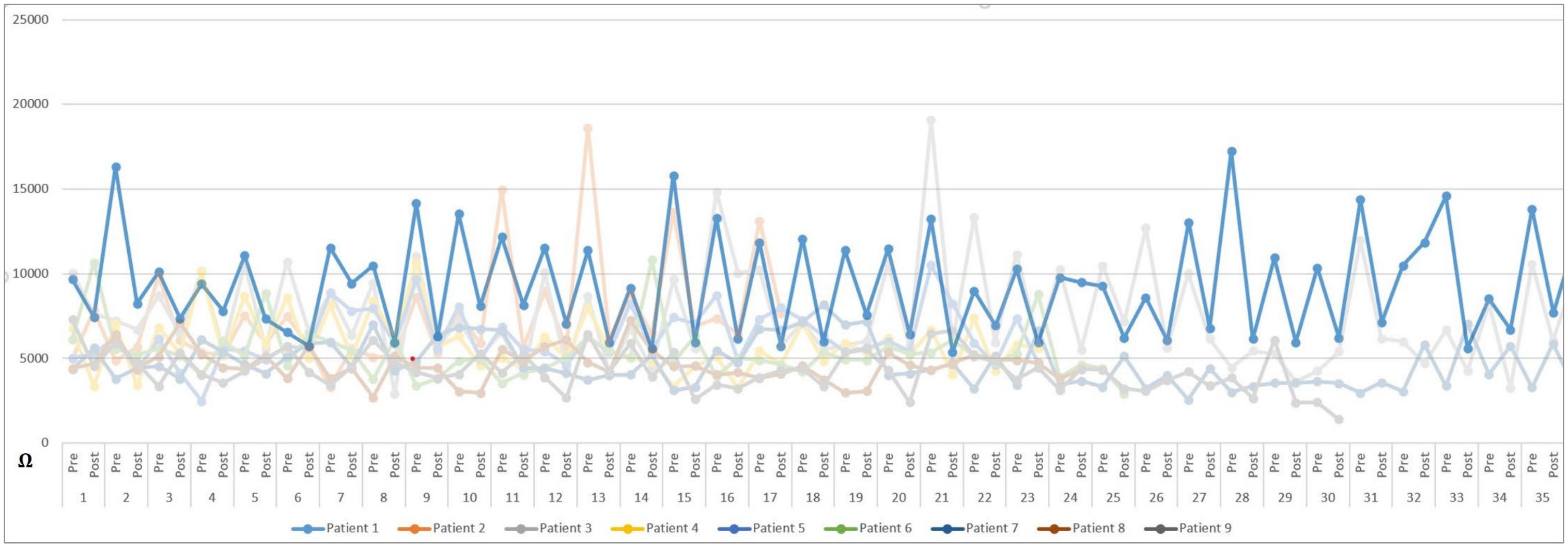

Figure S3. The results of galvanic response in pre- and post- each treatment
